# A Multiplex Polymerase Chain Reaction Assay for the Detection of Herpes Simplex Virus, Cytomegalovirus, and Varicella-Zoster Virus in Cerebrospinal Fluid

**DOI:** 10.3390/microorganisms13010111

**Published:** 2025-01-08

**Authors:** Tatjana Luzius, Samuel D. Jeske, Julia Baer, Uta Goelnitz, Ulrike Protzer, Jochen M. Wettengel

**Affiliations:** 1 School of Medicine and Health, Institute of Virology, Technical University of Munich/Helmholtz Munich, 81675 Munich, Germanyprotzer@tum.de (U.P.); 2 German Center for Infection Research (DZIF), Munich Partner Site, 81675 Munich, Germany; 3 QIAGEN GmbH, Strategic Lab Consultancy, 40724 Hilden, Germany

**Keywords:** multiplex PCR, viral meningitis, clinical virology

## Abstract

Viral meningitis poses a significant clinical challenge due to its rapid onset and potential progression to life-threatening encephalitis. Early detection of treatable viral pathogens such as Herpes simplex virus (HSV), Cytomegalovirus (CMV), and Varicella-zoster virus (VZV) is essential for initiating appropriate therapies. However, multiplex PCRs for the rapid and simultaneous detection of these pathogens are scarce due to the complex PCR design and the elaborate validation process using cerebrospinal fluid samples. In this study, we established and validated a novel multiplex PCR assay for detecting HSV, CMV, and VZV in cerebrospinal fluid samples and implemented the assay on a fully automated platform.

## 1. Introduction

Meningitis poses a significant challenge for patients due to its rapid onset and potential progression to encephalitis, which can be lethal. Clinically, meningitis can present with a wide range of symptoms, including headache, stiff neck, fever, nausea, and heightened sensitivity to light or sound [1]. The severity and duration of these symptoms can vary, with acute cases resolving in less than five days, while chronic meningitis may persist for over 30 days [2,3].

The etiological agents that are responsible for most acute infectious meningitis cases can be categorized into bacterial and viral pathogens. *Neisseria meningitidis*, *Streptococcus pneumoniae*, *Haemophilus influenzae*, and group B streptococcus such as *Streptococcus agalactiae* or *Streptococcus pyogenes* are recognized as the predominant bacterial pathogens [4]. Viral meningitis is mainly caused by enteroviruses, as well as Herpes simplex virus 1 (HSV-1) and Herpes simplex virus 2 (HSV-2) [5,6,7,8,9,10,11,12]. Other viral agents with lower incidence rates include Lymphocytic choriomeningitis virus (LCMV), Mumps virus (MuV), Cytomegalovirus (CMV), and Varicella-zoster virus (VZV) [11,12,13,14,15]. Additionally, several orthoflaviviruses can cause meningitis and encephalitis, including tick-borne encephalitis virus (TBEV), Powassan virus (PWV), Japanese encephalitis virus (JEV), and West Nile virus (WNV) [16,17,18,19,20,21,22,23].

Early and accurate diagnosis of meningitis is crucial for initiating appropriate therapeutic interventions [1,24,25]. However, this process is inherently challenging due to the diversity of causative pathogens and the symptomatic overlap with other neurological disorders. The standard diagnostic procedure involves a lumbar puncture and subsequent cerebrospinal fluid (CSF) analysis [13,26]. To detect bacterial infections in CSF, the standard methods include direct or pre-cultured microscopy of the samples using Gram staining and polymerase chain reactions (PCRs) to identify or confirm the respective bacterial pathogen [26]. In contrast, viral infections are typically detected only via PCR, as electron microscopy is not established and validated as a standard diagnostic method to reliably identify a specific virus [27,28,29,30].

A standard therapeutic regimen for bacterial meningitis may include administering third-generation cephalosporins (such as ceftriaxone or cefotaxime) in combination with ampicillin or vancomycin for approximately 7–14 days [26]. Treatment of viral meningitis, however, is more complicated, since directly acting antivirals are scarce. So far, viral meningitis caused by HSV-1, HSV-2, CMV, and VZV has the most established and effective standard therapy [31,32,33].

For HSV-induced meningitis, acyclovir can be used as the antiviral of choice, typically administered intravenously for 10–14 days [33]. CMV meningitis can be treated with ganciclovir or valganciclovir, often in combination with foscarnet for severe cases [31,34]. VZV meningitis can be managed with acyclovir or valacyclovir, with varying treatment durations based on the severity of the infection [35,36].

Although determining the exact cause of viral meningitis is crucial for further treatment, accurate identification necessitates implementing multiple distinct PCR assays or novel approaches like next-generation sequencing (NGS) [37]. This analysis is both time-consuming and costly, so initial diagnostic efforts should be prioritized to identify treatable viral infections caused by HSV-1, HSV-2, CMV, and VZV.

Numerous individual PCR protocols for detecting these pathogens have been established [38,39,40,41]. However, integrating these individual reactions into a multiplex approach necessitates the rational design of each primer and probe sequence to ensure that the individual PCR can operate under a shared temperature profile without interfering with the others. This guarantees both the specificity and sensitivity of the multiplex PCR approach. Consequently, there are only a few protocols for a multiplex PCR for HSV-1, HSV-2, CMV, and VZV, typically for detecting the pathogens in ocular or tear fluid in cases of clinical suspicion of acute uveitis [28,42,43].

Validated multiplex PCR assays for analyzing complex materials, such as CSF, are rare, or the individual primer sequences for adaptation on other platforms, such as automated random-access analyzers, are unavailable.

Here, we established and validated a novel multiplex PCR for detecting HSV-1, HSV-2, CMV, and VZV in CSF. We implemented this multiplex PCR on a fully automated system, the QIAGEN NeuMoDx288 Molecular System, to enable rapid diagnostics without extensive sample preparation. Designing and adapting the primer sequences allows for the use of standard PCR settings by maintaining both specificity and sensitivity.

## 2. Materials and Methods

### 2.1. Sample Collection and Preparation of Positive CSF Samples

All CSF samples were collected at the Institute of Virology of the Technical University of Munich. These samples were received for diagnostic purposes from various departments of the Klinikum Rechts der Isar of the Technical University of Munich.

The samples were analyzed for neurotropic viruses during routine diagnostics, as requested by the treating physicians. For these analyses, the Allplex Meningitis Panel Assay (which includes EBV, HHV-6, HHV-7, HSV-1, HSV-2, CMV, and VZV) and V2 (which includes Human Adenovirus, Enterovirus, Human Parechovirus, Mumps virus, and Parvovirus B19) (Seegene Inc., Seoul, Republic of Korea) were utilized, following the manufacturer’s instructions. In short, 200 µL of the CSF sample were extracted using the STARMag 96 ProPrep Kit (Seegene Inc., Seoul, Republic of Korea) to approximately 100 µL, and subsequently, 5 µL were used for the PCR.

When the test results were negative, the samples were aliquoted, pseudonymized, and preserved as negative CSF samples at 4 °C. Conversely, when the test results were positive, the samples were aliquoted, pseudonymized, and preserved as positive CSF samples from patients with clinically diagnosed meningitis at 4 °C.

Samples that tested positive for HSV-1, HSV-2, and VZV were obtained from swab samples collected at the Institute of Virology of the Technical University of Munich. These samples were received for diagnostic purposes from various departments of the Klinikum Rechts der Isar of the Technical University of Munich.

The positive CMV sample was derived from an amniotic fluid sample collected at the Institute of Virology of the Technical University of Munich. This sample was received for diagnostic purposes from various departments of the Klinikum Rechts der Isar of the Technical University of Munich.

To obtain positive samples for establishing and validating the assay, the viral transport medium of the swab samples or the amniotic fluid was initially diluted 1:100 in the negative CSF samples to create defined highly positive CSF samples (Ct < 30). These highly positive samples were further diluted 10- to 1000-fold based on the virus concentration to produce low-level positive CSF samples (Ct > 30).

### 2.2. Extraction of Viral Nucleic Acid

#### 2.2.1. Standard Method for the Extraction of Viral Nucleic Acid

The STARMag 96 ProPrep Kit (Seegene Inc., Seoul, Republic of Korea) served as the standard method for nucleic acid extraction from CSF samples. The extraction was performed utilizing the SEEPREP32 device (Seegene Inc., Seoul, Republic of Korea), following the manufacturer’s instructions. In short, 200 µL of the sample was mixed thoroughly with 10 µL of a proteinase K solution and 10 µL of the internal control in the first well of the reagent cartridge. The lysis mix was then incubated for 5 min at room temperature. After the incubation, 650 µL of the mixture was transferred into the second well of the reagent cartridge and mixed to homogenize the reagent mix. The extraction program for automated nucleic acid extraction was then executed on the SEEPREP32.

After the extraction process was completed, the eluates (~100 μL) were transferred to a 1.5 mL reaction tube and used for the specific PCR or stored at −20 °C until further use.

#### 2.2.2. Fully Automated Method for the Extraction of Viral Nucleic Acid

To establish the Laboratory-Developed Test (LDT), the fully automated extraction process was performed on the NeuMoDx288 system (QIAGEN, Hilden, Germany) utilizing the lysis buffer Lysis Buffer 2 (QIAGEN, Hilden, Germany). The sample volume for the extraction was set to 200 µL. The reaction conditions for the extraction were set to 50 °C for 10 min. After the extraction process was completed, the eluates (20 μL) were transferred to a 1.5 mL reaction tube and used for the specific PCR or stored at −20 °C until further use.

### 2.3. Performing Probe-Based qPCRs on the Fully Automated Platform

The probe-based qPCRs were conducted on the NeuMoDx platform (QIAGEN, Hilden, Germany) to validate the LDT. A 4 μL mixture of forward and reverse primers, as well as the hydrolysis probe(s), was added to each well of the NeuMoDx LDT primer/probe strip (QIAGEN, Hilden, Germany) following the manufacturer’s instructions and the determined probe concentration of 1.5 µM. For the PCR mix, the NeuMoDx LDT RNA Master Mix (QIAGEN, Hilden, Germany) was used.

The NeuMoDx-LDT Assay definition file (ADF) was modified specifically from the preinstalled template LDT QUAL-RNA according to the following parameters: Ct calling algorithm, second derivative; result type, qualitative; specimen type, CSF; specimen aspirate volume (μL), 200; specimen mix volume (μL), 600; lysis conditions, 600 s; lysis buffer, 2, 50 °C (medium); target, SPC2 (sample process control); reporter, yellow (530/555); peak minimum cycle, 28; peak maximum cycle, 34; minimum endpoint fluorescence, 1000; minimum peak height, 100; target, HSV; reporter, FAM (BHQ1), green (470/510); peak minimum cycle, 10; peak maximum cycle, 50; minimum endpoint fluorescence, 1000; minimum peak height, 100; target, CMV; reporter, Atto565 (BHQ2), orange (585/610); peak minimum cycle, 10; peak maximum cycle, 50; minimum endpoint fluorescence, 1000; minimum peak height, 100; target, VZV; reporter, Atto647N (BHQ2), far red (680/715); peak minimum cycle, 10; peak maximum cycle, 50; minimum endpoint fluorescence, 1000; minimum peak height, 100; PCR stage, reverse transcription (hold, 900 s, 50 °C); PCR stage, inactivation (hold, 240 s, 95 °C); PCR stage, cycle (cycle, 50 cycles); step denature, 6 s, at 95 °C, no detection; and step anneal, 19 s, at 60 °C, detection.

The PCR conditions for the LDT assay are presented in Table 1.

### 2.4. Performing Digital PCR for the Determination of Viral Load in Prepared Positive CSF Samples

A digital PCR (dPCR) was conducted to determine the viral loads of the respective positive CSF samples utilizing the QIAcuity One 5plex (QIAGEN). Nucleic acids from the starting sample were isolated using the STARMag 96 ProPrep Kit (Seegene) and subsequently used as a template for the dPCR. After evaluating the dPCR results, samples with a Ct value of at least 22 were selected. Subsequently, a mix for the dPCR was prepared with the QIAcuity Probe PCR Kit (QIAGEN), with primer concentrations of 1.67 µM and a probe concentration of 0.4 µM according to the manufacturer’s instructions. In short, a volume of 10 μL of this mixture was added to each tube in an 8-strip PCR tube. Subsequently, 2 μL of the respective DNA template was pipetted into the mixture. The PCR strips were gently agitated to homogenize the contents, and the prepared mixture with the DNA template (PCR mix) was briefly centrifuged. Next, 11 μL of the PCR mix was carefully pipetted into the wells of the QIAcuity Nanoplate 8.5k (QIAGEN), which was positioned on the plate holder. To prevent bubble formation, the pipette tips were gently placed at the bottom of the wells, and only the first stop of the pipette was utilized. Finally, the sealing film provided with the plate was applied, ensuring that all wells were adequately covered, and no bubbles were visible beneath the film. A film roller was employed to ensure that the film was properly adhered to the plate.

The dPCR conditions are described in Table 2.

### 2.5. Analysis of the Limit of Detection (LoD)

The limit of detection (LoD) (95% confidence interval) was determined by analyzing the previously generated highly positive CSF samples for HSV-1, HSV-2, VZV, and CMV at decreasing concentrations. The LoD was subsequently calculated using probit analysis (MedCalc Software Ltd., version 23.0.5, Ostend, Belgium).

## 3. Results

### 3.1. Generation of Consensus Sequences and Primer Design

For the individual design of specific primers and probes, consensus sequences for HSV-1, HSV-2, CMV, and VZV were generated (Supplemental Figures S1–S3). For this, a randomized subset of sequences from the available whole genomes of each virus was obtained from the NCBI Virus database (HSV-1: 91; HSV-2: 113; CMV: 181; and VZV: 169) [44]. The sequences were aligned using the MAFFT algorithm, and a consensus sequence at 95% agreement was generated. The consensus sequences and a graphical overview of the conserved regions are provided in the Supplementary Materials.

Potential primer and probe sequences were selected according to the standard recommendations. Highly conserved regions were screened for potential amplicon lengths of 70–150 bp, avoiding regions with high GC contents or potential secondary structures to minimize the risk of impaired amplification. All sequences were chosen with melting temperatures (Tm) of approximately 58–60 °C for forward and reverse primers and probes to have Tm values that were 8–10 °C higher to ensure a simultaneous amplification of all individual reactions in the multiplex approach. Sequences were also screened and optimized to prevent dimer formation and self-complementarity and checked against publicly available genomic databases to confirm their specificity, with only minimal homology with non-target sequences. In silico validation was conducted to simulate PCR conditions and predict primer performance, allowing for optimization before empirical testing. In the last step, primers and probes were selected to have no or only minimal cross-reactivity with the respective other primers, and probes and checked for optimal annealing to the generated consensus sequences (Table 3).

### 3.2. Optimization of Probe Concentration

After successfully designing the individual primers and probes, each reaction was optimized for performance on a fully automated platform. Negatively tested CSF samples were spiked with the corresponding virus at concentrations exceeding 4000 viral particles/mL, and individual PCR assays were conducted. Fluorescence was measured for each reaction by varying the probe concentration while maintaining a constant primer concentration (Supplemental Figures S4, S6, S8 and S10). The optimal probe concentration for all primers was determined to be between 1 µM and 2 µM, with a final concentration set at 1.5 µM (Supplemental Figures S5, S7, S9 and S11).

### 3.3. Multiplexing of the Individual PCR Assays

**Figure 1:**
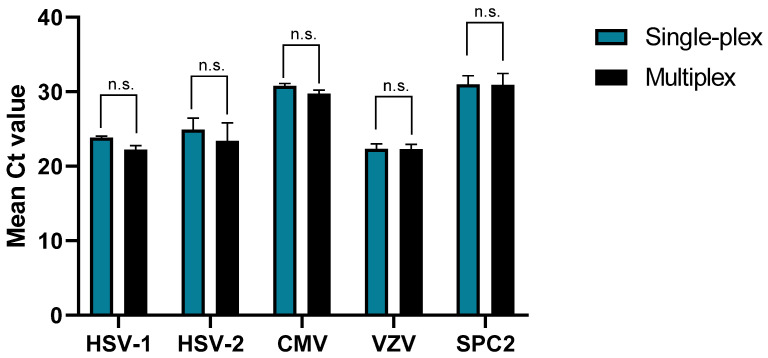
Comparison of the assay in a single-plex (blue) and multiplex (black) approach. Spiked CSF samples with variable virus concentrations of HSV-1, HSV-2, CMV, and VZV were analyzed in a single detection assay versus the multiplex assay (*N* = 4). Bars and whiskers indicate the mean Ct and the standard deviation. For statistical analysis, a two-way ANOVA with Bonferroni correction was performed. n.s.: not significant.

In the next step, the performance of each PCR assay was determined in the context of individual PCR assays (single-plex) and a multiplex approach, including all primer/probe pairs, to assess the impact of multiplexing on the PCR performance. For this purpose, CSF samples were spiked with HSV-1, HSV-2, CMV, and VZV and measured both in a single-plex and multiplex approach. All measured samples yielded a positive signal only for the respective virus, while the other PCR reactions showed no signal, indicating no cross-reactivity due to multiplexing. The direct comparison of the Ct values demonstrated that multiplexing only had a minimal impact on the Ct values, with a difference of less than 2 Ct values (Figure 1).

### 3.4. Assessment of Performance

To assess the performance of the multiplex PCR, the assay was validated for intra-assay and inter-assay precision, as well as specificity and sensitivity.

**Figure 2:**
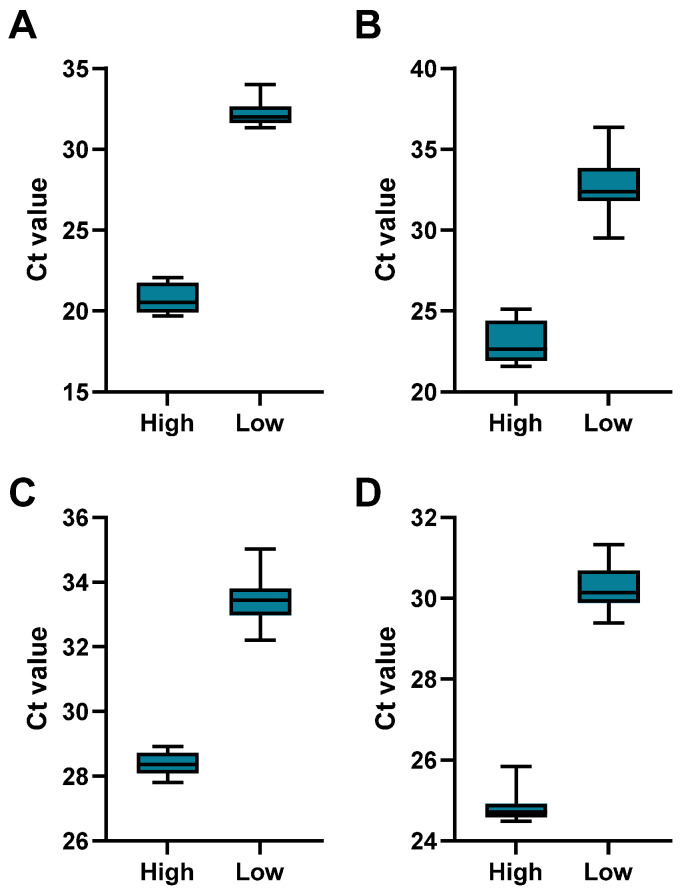
Intra-assay performance of the Meningitis-LDT-PCR. Spiked CSF samples with variable virus concentrations of (**A**) HSV-1, (**B**) HSV-2, (**C**) CMV, and (**D**) VZV were analyzed. Precision was analyzed using samples with a high or low virus concentration for each virus (*N*  =  12). The box plots show the median, interquartile ratio (box), and minimum to maximum (whiskers).

First, the intra-assay variability was analyzed by testing individual CSF samples that were spiked with the corresponding virus at low (Ct > 30) and high (Ct < 30) concentrations in replicates (*N* = 12 per concentration and virus). The results (Figure 2, Table 4) demonstrated acceptable coefficients of variation, ranging from 1.20% to 5.47% for all viruses, indicating that our assay is both reliable and consistent within a single run.

**Figure 3:**
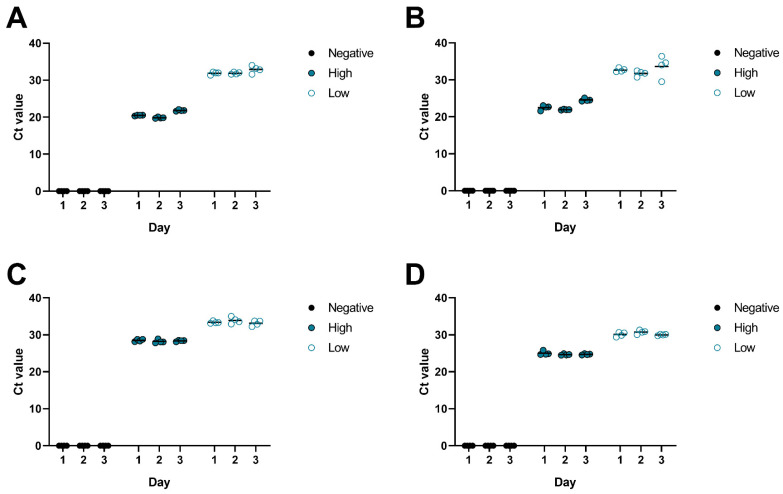
Analysis of intra- and inter-assay performance by using HSV-1- (**A**), HSV-2- (**B**), CMV- (**C**), and VZV (**D**)-negative and -positive samples with high and low viral loads (*N* = 4 per day). Negative results are indicated with a Ct value of 0. The mean is shown as a black line.

Next, the multiplex PCR was performed on negative CSF samples, and, as before, CSF samples spiked with the corresponding virus at low (Ct > 30) and high (Ct < 30) concentrations in replicates (*N* = 4 per concentration and virus) over three consecutive days to analyze the inter-assay precision. All negative samples tested negative on all days. The results of the positive samples (Figure 3, Table 5) demonstrated acceptable coefficients of variation, ranging from 0.51% to 6.06% for all viruses, indicating consistent performance across different runs.

To analyze the specificity of our assay, the cross-reactivity was tested with other bacterial and viral pathogens that can cause meningitis. Negatively tested CSF samples (*N* = 4 per pathogen) were spiked with *Streptococcus pneumoniae*, *Neisseria meningitidis*, *Haemophilus influenzae*, Human immunodeficiency virus (HIV), Enterovirus, Epstein–Barr virus (EBV), Human herpesvirus 6 (HHV-6), and Human herpesvirus 7 (HHV-7) and tested with our multiplex PCR. All samples tested negative for HSV-1, HSV-2, CMV, and VZV, demonstrating no cross-reactivity and confirming the specificity of our assay.

**Figure 4:**
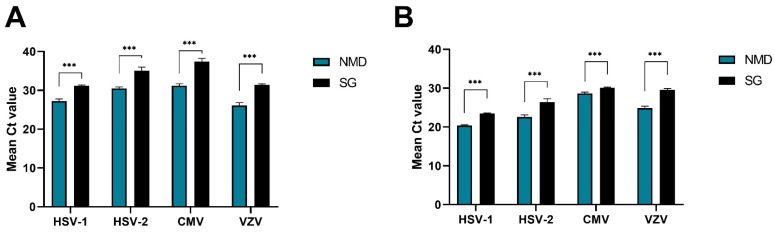
Comparison of methods. The samples were prepared using the established method in the laboratory for detecting neurotropic viruses by Seegene (SG, black) and with the newly established Meningitis-LDT assay, performed on the fully automated random-access platform NeuMoDx by QIAGEN (NMD, blue) with *N* = 4. (**A**) Results for CSF samples with low viral loads of each virus. (**B**) Results for high viral load samples of each virus (two-tailed unpaired *t*-test: ***: *p* < 0.001).

Subsequently, our multiplex assay was compared to a commercially available assay with high and low positive CSF samples. We found consistent results across both assays, with our assay showing lower Ct values when compared to the commercial assay (Figure 4).

**Figure 5:**
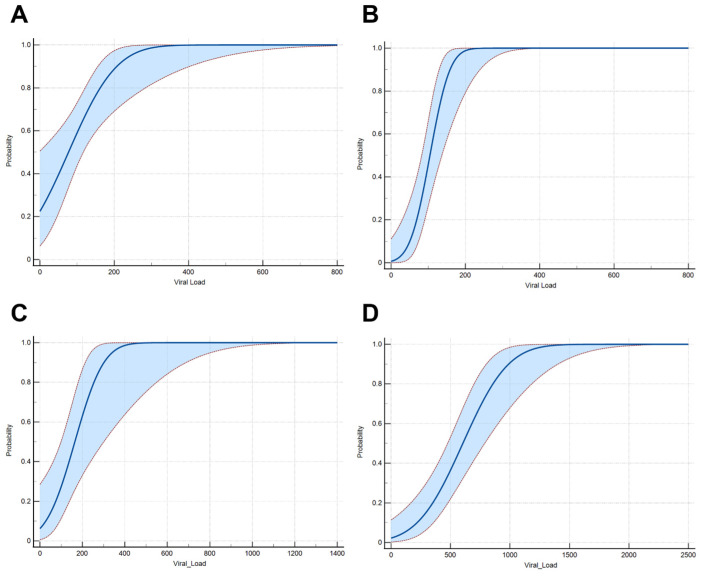
Dose–Response plot for determining the limit for 95% probability of detection for (**A**) HSV-1, (**B**) HSV-2, (**C**) CMV, and (**D**) VZV. The analysis was carried out by using the probit model.

In the final step, the sensitivity of our assay was analyzed by spiking CSF samples with HSV-1, HSV-2, CMV, and VZV and quantifying the viral concentrations via dPCR. A serial dilution was generated, and each dilution was tested multiple times with our assay. Our results show that the multiplex assay has a LoD (95%) for HSV-1 of 244 viral particles/mL (95% CI: [181–504 particles/mL]), HSV-2 of 174 viral particles/mL (95% CI: [141–271 particles/mL]), CMV of 340 viral particles/mL (95% CI: [239–805 particles/mL]), and VZV of 1098 viral particles/mL (95% CI: [883–1588 particles/mL]) (Figure 5).

Overall, our multiplex PCR assay demonstrates high precision, specificity, and sensitivity, making it a reliable tool for the detection of viral pathogens in CSF samples.

### 3.5. Clinical Performance

Following the successful determination of the assay’s performance, we validated the assay using patient-derived clinical samples. This clinical performance assessment was conducted with previously identified positive CSF samples with diagnosed meningitis, including one HSV-1-positive CSF sample and four VZV-positive ones. The tests for the positive CSF samples were originally conducted with the commercially available Allplex Meningitis Panel Assay V1 (control device). We re-tested these samples with our multiplex Meningitis-LDT assay (test device) and could detect a 100% agreement with the original result with considerably lower Ct values (Table 6). Positive samples for CMV and HSV-2 were not available and therefore were not tested.

## 4. Discussion

Early and accurate detection and identification of the corresponding pathogen(s) are essential for the effective treatment of meningitis. While most bacterial meningitis can be treated with specific antibiotics, there are only limited antiviral options available for treating viral meningitis. Notably, cases of viral meningitis caused by HSV, CMV, and VZV have the most established and effective standard therapies [31,33,34,36]. From a clinical perspective, it is, therefore, beneficial to prioritize testing for these viral pathogens.

The availability of multiplex PCR assays for detecting these viruses in CSF is highly limited, particularly in the context of automated random-access platforms [45,46]. However, most of these platforms offer the flexibility to implement self-developed or external PCR assays, combining the advantages of minimal hands-on time, a short turnaround time, high throughput, and, most importantly, on-demand testing with individually designed single-plex or multiplex assays.

Here, we describe the establishment and validation of a multiplex PCR assay for detecting HSV-1, HSV-2, CMV, and VZV, intended for use on fully automated random-access platforms.

After generating consensus sequences and designing pathogen-specific primers and probes for HSV-1, HSV-2, CMV, and VZV, we optimized the probe concentrations to ensure consistent performance. Furthermore, our results demonstrated that the assay could reliably detect each virus without cross-reactivity of each individual assay, even when multiple pathogens were present in the sample.

The performance of the multiplex PCR assay also showed high intra-assay and inter-assay precision, with consistent CV% indicating a reliable performance in single or across different runs, even though CSF is a complex sample type [47,48,49].

The assay’s specificity was confirmed by testing against other bacterial and viral pathogens that can cause meningitis, with no cross-reactivity observed. This high specificity is essential for ensuring that the detected pathogens are indeed the causative agents of the infection.

In the final step, the specificity of the assay was evaluated using samples with specific virus concentrations, determined by dPCR. The results revealed LoDs for HSV-1, HSV-2, CVV, and VZV of 244, 174, 340, and 1098 viral particles/mL, respectively. Interestingly, the LoD values for HSV-1, HSV-2, and CMV fall within a sensitive range of IVD-certified and commercially available assays [28,29,45,46,50]. In contrast, the LoD for VZV was determined to be 1098 viral particles/mL. This relatively high LoD for a diagnostic PCR could lead to false negative VZV results, especially in patients with low viral titers. Interestingly, this VZV-specific LoD is similar to many published single-plex assays, as well as multiplex assays, suggesting a general difficulty in detecting VZV in CSF and emphasizing the need for individual optimization of the sample extraction process on the respective instrument [41,51,52,53]. Therefore, for individual use, it is recommended to conduct a corresponding validation to assess the platform-specific inter- and intra-assay precision, and especially sensitivity, before implementing this assay into routine diagnostics.

In summary, this multiplex PCR assay allows for individual adaptation and implementation, tailored to the specific laboratory equipment, particularly for random-access platforms, ensuring rapid and precise detection of HSV-1, HSV-2, CMV, and VZV from CSF.

## Figures and Tables

**Table 1:** PCR reaction conditions for the performance of the assay on the NeuMoDx platform.

Step	Cycles	Duration	Temperature
		15 min	50 °C
Initial activation	1	4 min	95 °C
Denaturation	50	6 s	95 °C
Annealing		19 s	60 °C

**Table 2:** dPCR conditions.

Step	Cycles	Duration	Temperature
Initial activation	1	2 min	95 °C
1. PCR Step	40	15 s	95 °C
		1 min	60 °C
2. PCR Step	5	15 s	95 °C
		1 min	60 °C
Cooling	1	30 s	40 °C

**Table 3:** Primer (Fw, Rv) and probe (So) sequences for detecting HSV, CMV, and VZV.

Primer/Probe	Sequence	5′Modification	3′Modification
T-HSV-Fw	CCTGGAGGTGCGGTTGATAA	-	-
T-HSV-Rv	AGAAAAAGTACATCGGCGTCATCT	-	-
T-HSV-So	CCAGATCCACGCCCTTGATGAGCAT	FAM	BHQ1
T-CMV-2-Fw	GCGGTTCGGGCACTAGTTC	-	-
T-CMV-2 Rv	CAGCGCAGCTACTTTTACTGTGA	-	-
T-CMV So	CAATGACCTCACGCAGCCTATCGGTG	ATTO565	BHQ2
T-VZV Fw	CAGTACRTTGCATAACCTGTCCAT	-	-
T-VZV Rv	GCCACGATCCCGGAGAA	-	-
T-VZV So	CATTTTCAGTTGCGCGGACGCC	ATTO647N	BHQ2

**Table 4:** Analysis of intra-assay performance for HSV-1, HSV-2, CMV, and VZV.

Analyte	Concentration	Minimum	Maximum	Range	Mean	Std. Deviation	CV%
HSV-1	Low	31.34	34.00	2.66	32.22	0.76	2.36
	High	19.69	22.08	2.39	20.72	0.87	4.21
HSV-2	Low	29.50	36.37	6.87	32.67	1.79	5.47
	High	21.59	25.13	3.54	23.00	1.25	5.43
CMV	Low	32.20	35.03	2.83	33.47	0.71	2.12
	High	27.81	28.92	1.11	28.37	0.34	1.20
VZV	Low	29.39	31.33	1.94	30.28	0.55	1.81
	High	24.48	25.84	1.36	24.81	0.36	1.44

**Table 5:** Analysis of inter-assay performance for HSV-1, HSV-2, CMV, and VZV.

Analyte	Concentration	Mean Day 1	Mean Day 2	Mean Day 3	Range of Means	Grand Mean	Std. Deviation of Means	CV%
HSV-1	Low	31.88	31.89	32.90	1.02	32.22	0.59	1.82
	High	20.48	19.85	21.83	1.98	20.72	1.01	4.88
HSV-2	Low	32.66	31.73	33.62	1.89	32.67	0.95	2.89
	High	22.49	21.94	24.58	2.64	23.00	1.39	6.06
CMV	Low	33.39	33.90	33.13	0.77	33.47	0.39	1.17
	High	28.51	28.22	28.38	0.29	28.37	0.15	0.51
VZV	Low	30.10	30.74	30.00	0.74	30.28	0.40	1.33
	High	25.04	24.66	24.73	0.38	24.81	0.20	0.81

**Table 6:** Assessment of the clinical performance of the multiplex assay.

Sample	Virus	Ct (Control Device—Allplex Meningitis Panel Assay V1)	Ct (Test Device—Meningitis-LDT)
1	HSV-1	29.45	24.11
2	VZV	29.00	25.45
3	VZV	27.05	21.44
4	VZV	33.16	26.06
5	VZV	33.54	26.51

## Data Availability

The raw data presented in this study are available on request from the authors.

## Supplementary Materials

The following supporting information can be downloaded at https://www.mdpi.com/article/10.3390/microorganisms13010111/s1: Figure S1: (A) Generated consensus sequences for HSV-1 and HSV-2 were used to create a combined HSV-1xHSV-2 consensus sequence. (B) Alignment of the used primers and probes to the combined consensus of HSV-1xHSV-2; Non-conserved regions N are marked in yellow, Figure S2: (A) Generated consensus sequence for CMV. (B) Alignment of primers and probes to the consensus of CMV; Non-conserved regions N are marked in yellow, Figure S3: (A) Generated consensus sequence for VZV. (B) Alignment of primers and probes to the consensus of VZV; Non-conserved regions N are marked in yellow, Figure S4: Optimization of the probe concentration for the detection of HSV-1 using concentrations from 0.125 µM to 16 µM. The concentration for the forward and reverse primers was 1.8 µM for every dilution. (A) Plot of the mean Ct values of duplicates as a function of probe concentration; The error bars correspond to the standard deviation of the duplicates. (B) Plot of the fluorescence curves as a function of the PCR cycle. N/A: No amplification, Figure S5: Further optimization of the probe concentration for the detection of HSV-1 for concentrations from 1 μM to 2 μM at two annealing temperatures 58 °C and 60 °C. (A) Comparison of the mean Ct values for the different probe concentrations at the two annealing temperatures. The error bars correspond to the standard deviation of duplicates. (B) Plot of the fluorescence curves as a function of the PCR cycle for 58 °C. (C) Representation of the fluorescence curves as a function of the PCR cycle for 60 °C, Figure S6: Optimization of the probe-concentration for the detection of HSV-2 using concentrations from 0.125 µM to 16 µM. The concentration for the forward and reverse primers was 1.8 µM for every dilution. (A) Plot of the mean Ct values of duplicates as a function of probe concentration; The error bars correspond to the standard deviation of the duplicates. (B) Plot of the fluorescence curves as a function of the PCR cycle. N/A: No amplification, Figure S7: Further optimization of the probe concentration for the detection of HSV-2 for concentrations from 1 μM to 2 μM at two annealing temperatures 58 °C and 60 °C. (A) Comparison of the mean Ct values for the different probe concentrations at the two annealing temperatures. The error bars correspond to the standard deviation of duplicates. (B) Plot of the fluorescence curves as a function of the PCR cycle for 58 °C. (C) Representation of the fluorescence curves as a function of the PCR cycle for 60 °C, Figure S8: Optimization of the probe-concentration for the detection of CMV using concentrations from 0.125 µM to 16 µM. The concentration for the forward and reverse primers was 1.8 µM for every dilution. (A) Plot of the mean Ct values of duplicates as a function of probe concentration; The error bars correspond to the standard deviation of the duplicates. (B) Plot of the fluorescence curves as a function of the PCR cycle. N/A: No amplification, Figure S9: Further optimization of the probe concentration for the detection of CMV for concentrations from 1 μM to 2 μM at two annealing temperatures 58 °C and 60 °C. (A) Comparison of the mean Ct values for the different probe concentrations at the two annealing temperatures. The error bars correspond to the standard deviation of duplicates. (B) Plot of the fluorescence curves as a function of the PCR cycle for 58 °C. (C) Representation of the fluorescence curves as a function of the PCR cycle for 60 °C, Figure S10: Optimization of the probe-concentration for the detection of VZV using concentrations from 0.125 µM to 16 µM. The concentration for the forward and reverse primers was 1.8 µM for every dilution. (A) Plot of the mean Ct values of duplicates as a function of probe concentration; The error bars correspond to the standard deviation of the duplicates. (B) Plot of the fluorescence curves as a function of the PCR cycle. N/A: No amplification, Figure S11: Further optimization of the probe concentration for the detection of VZV for concentrations from 1 μM to 2 μM at two annealing temperatures 58 °C and 60 °C. (A) Comparison of the mean Ct values for the different probe concentrations at the two annealing temperatures. The error bars correspond to the standard deviation of duplicates. (B) Plot of the fluorescence curves as a function of the PCR cycle for 58 °C. (C) Representation of the fluorescence curves as a function of the PCR cycle for 60 °C, Table S1: List of all sequences that were used to generate the consensus sequences for HSV-1, HSV-2, CMV and VZV.
