# Supplementary material for: A Multiplex Polymerase Chain Reaction Assay for the Detection of Herpes Simplex Virus, Cytomegalovirus, and Varicella-Zoster Virus in Cerebrospinal Fluid
Source: Microorganisms. 2025 Jan 8;13(1):111. doi: 10.3390/microorganisms13010111 (PMC11767304; doi:10.3390/microorganisms13010111)
Supplement: Supplementary file 1 [file microorganisms-13-00111-s001.zip › microorganisms-3344275-supplementary.pdf]

Supplementary Information

A Multiplex Polymerase Chain Reaction Assay for the Detection of Herpes Simplex Virus, Cytomegalovirus, and Varicella-Zoster Virus in Cerebrospinal Fluid

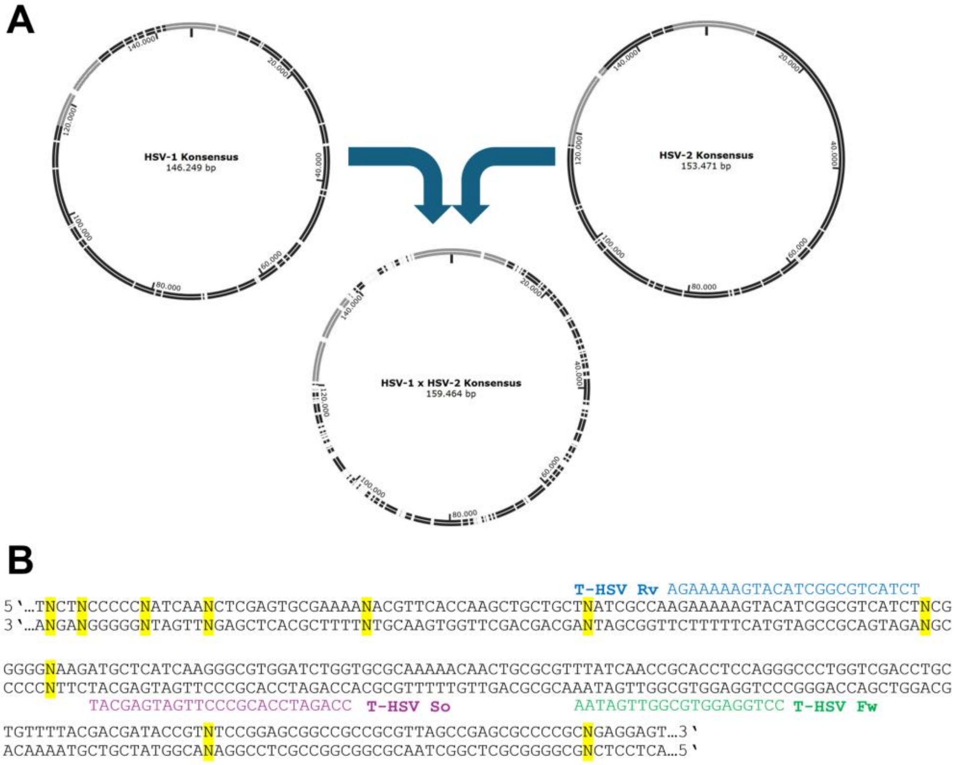

Figure S1. (A) Generated consensus sequences for HSV-1 and HSV-2 were used to create a combined HSV-1xHSV-2 consensus sequence. (B) Alignment of the used primers and probes to the combined consensus of HSV-1xHSV-2; Non-conserved regions N are marked in yellow.

A

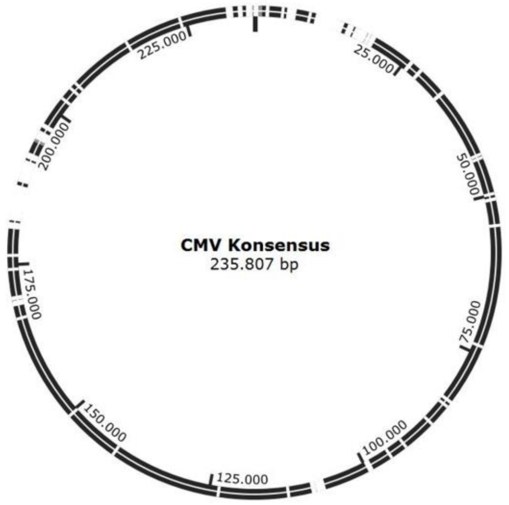

B

```
                        T-CMV-2 Fw GCGGTTCGGGCACTAGTTC    T-CMV So CAATGACCTCACGCAG
5'...GGCGTGACAGACACGGCGTATGGCGTCCGCGGTTCGGGCACTAGTTCGCCCACGCTGGCAATNACCTCACGCAG
3'...CCGCACTGTCTGTGCCGCATACCGCAGGCGCCAAGCCCGTGATCAAGCGGGTGCGACCGTTANTGGAGTGCGTC

CCTATCGGTG
CCTATCGGTGTCGCTGTACTCACAGTAAAAGTAGCTGCGCTGCCCGAAAACGTTGACGCAGATACTGTAGCCG...3'
GGATAGCCACAGCGACATGAGTGTCATTTTCATCGACGCGACGGGCTTTTGCAACTGCGTCTATGACATCGGC...5'
                       AGTGTCATTTTCATCGACGCGAC T-CMV-2 Rv
```

Figure S2. (A) Generated consensus sequence for CMV. (B) Alignment of primers and probes to the consensus of CMV; Non-conserved regions N are marked in yellow.

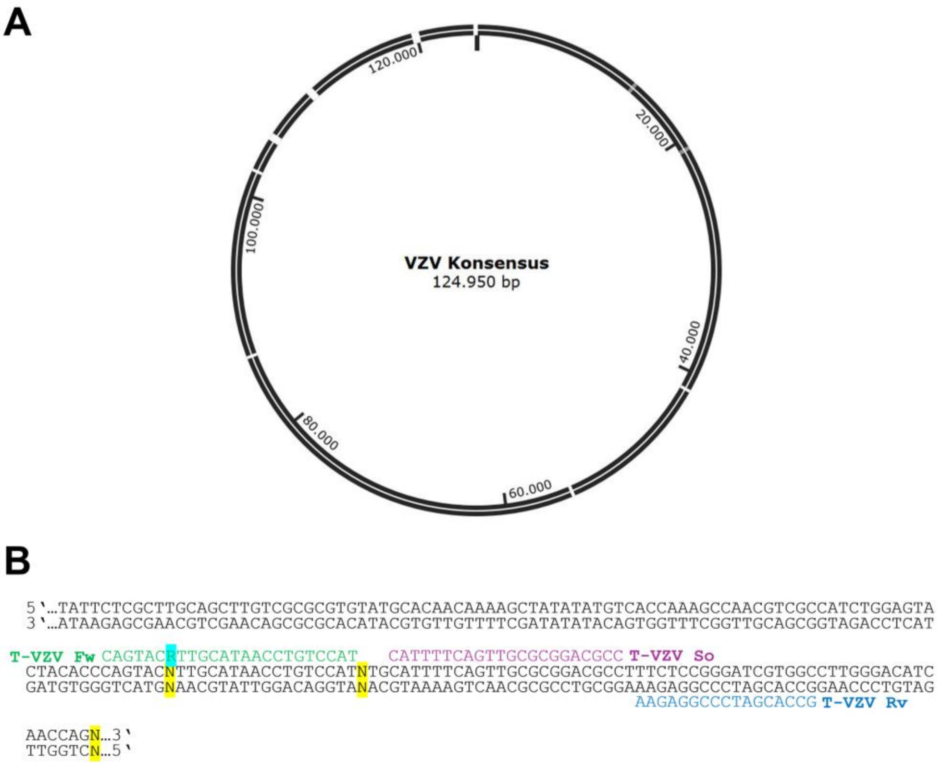

Figure S3. (A) Generated consensus sequence for VZV. (B) Alignment of primers and probes to the consensus of VZV; Non-conserved regions N are marked in yellow.

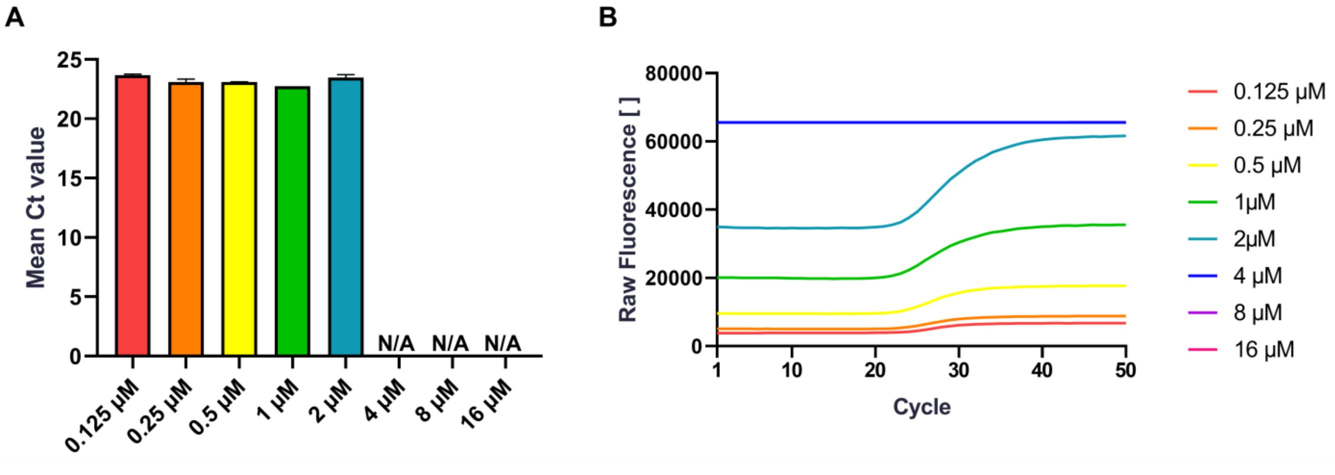

Figure S4. Optimization of the probe concentration for the detection of HSV-1 using concentrations from 0.125 µM to 16 µM. The concentration for the forward and reverse primers was 1.8 µM for every dilution. (A) Plot of the mean Ct values of duplicates as a function of probe concentration; The error bars correspond to the standard deviation of the duplicates. (B) Plot of the fluorescence curves as a function of the PCR cycle. N/A: No amplification.

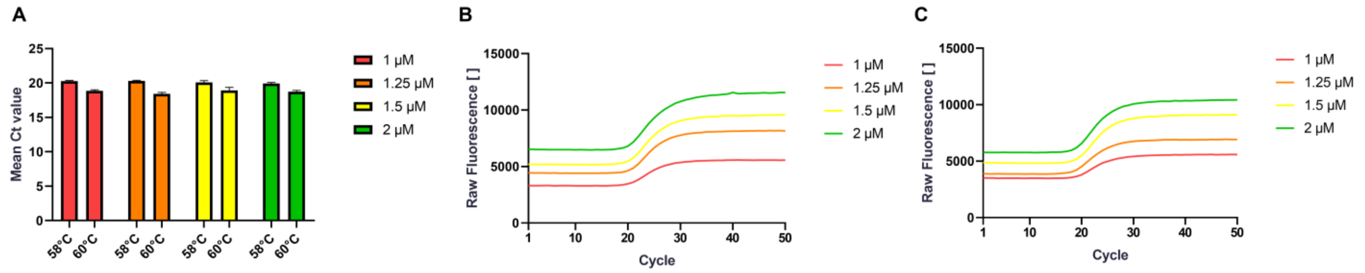

Figure S5. Further optimization of the probe concentration for the detection of HSV-1 for concentrations from 1 µM to 2 µM at two annealing temperatures 58°C and 60°C. (A) Comparison of the mean Ct values for the different probe concentrations at the two annealing temperatures. The error bars correspond to the standard deviation of duplicates. (B) Plot of the fluorescence curves as a function of the PCR cycle for 58°C. (C) Representation of the fluorescence curves as a function of the PCR cycle for 60°C.

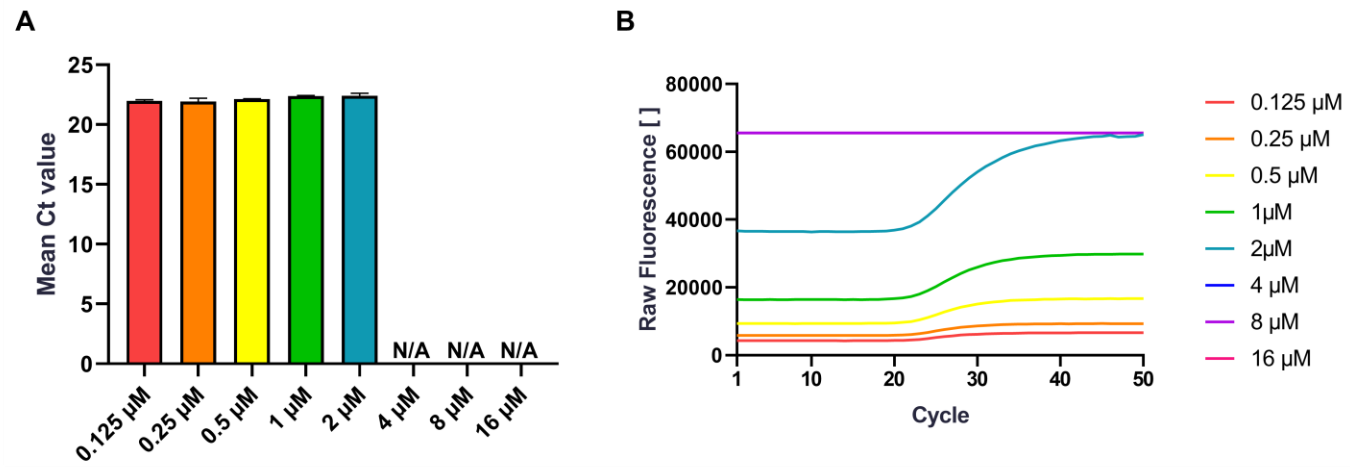

Figure S6. Optimization of the probe-concentration for the detection of HSV-2 using concentrations from 0.125 µM to 16 µM. The concentration for the forward and reverse primers was 1.8 µM for every dilution. (A) Plot of the mean Ct values of duplicates as a function of probe concentration; The error bars correspond to the standard deviation of the duplicates. (B) Plot of the fluorescence curves as a function of the PCR cycle. N/A: No amplification.

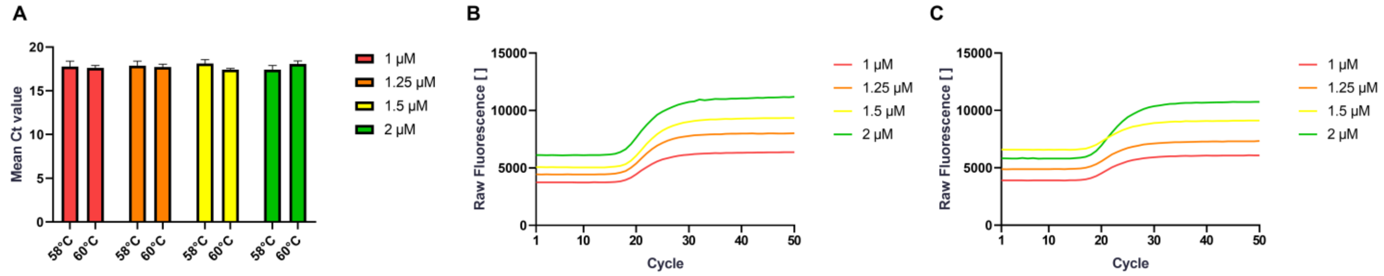

Figure S7. Further optimization of the probe concentration for the detection of HSV-2 for concentrations from 1 µM to 2 µM at two annealing temperatures 58°C and 60°C. (A) Comparison of the mean Ct values for the different probe concentrations at the two annealing temperatures. The error bars correspond to the standard deviation of duplicates. (B) Plot of the fluorescence curves as a function of the PCR cycle for 58°C. (C) Representation of the fluorescence curves as a function of the PCR cycle for 60°C.

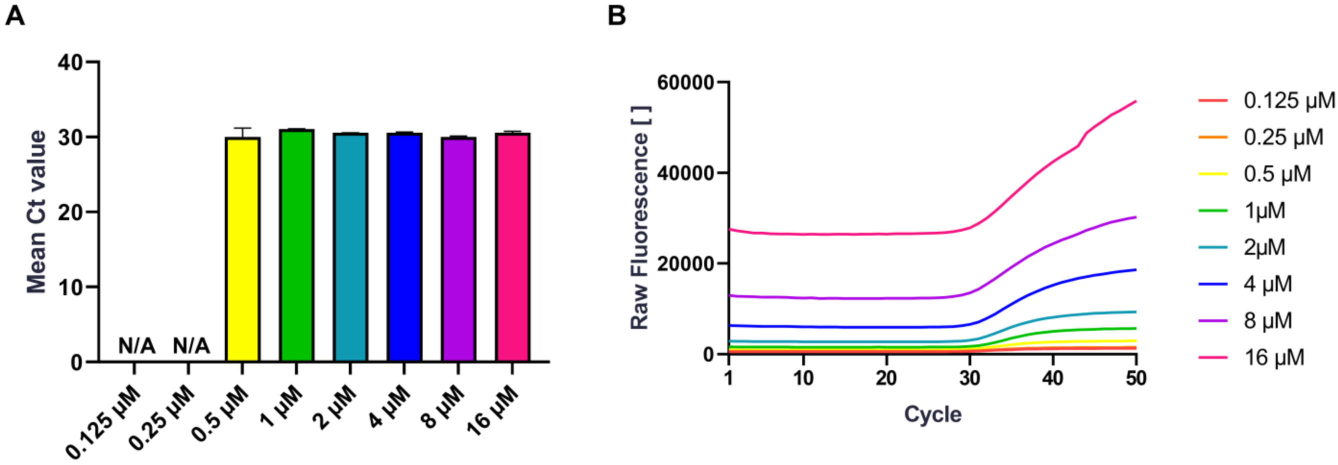

Figure S8. Optimization of the probe-concentration for the detection of CMV using concentrations from 0.125 µM to 16 µM. The concentration for the forward and reverse primers was 1.8 µM for every dilution. (A) Plot of the mean Ct values of duplicates as a function of probe concentration; The error bars correspond to the standard deviation of the duplicates. (B) Plot of the fluorescence curves as a function of the PCR cycle. N/A: No amplification.

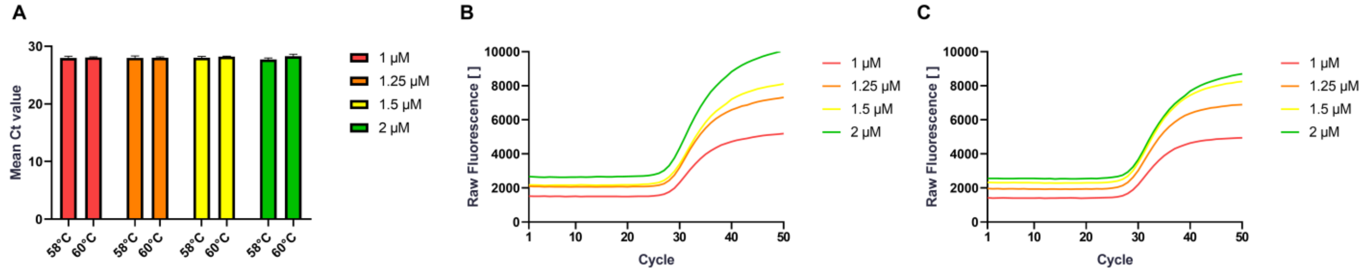

Figure S9. Further optimization of the probe concentration for the detection of CMV for concentrations from 1 µM to 2 µM at two annealing temperatures 58°C and 60°C. (A) Comparison of the mean Ct values for the different probe concentrations at the two annealing temperatures. The error bars correspond to the standard deviation of duplicates. (B) Plot of the fluorescence curves as a function of the PCR cycle for 58°C. (C) Representation of the fluorescence curves as a function of the PCR cycle for 60°C.

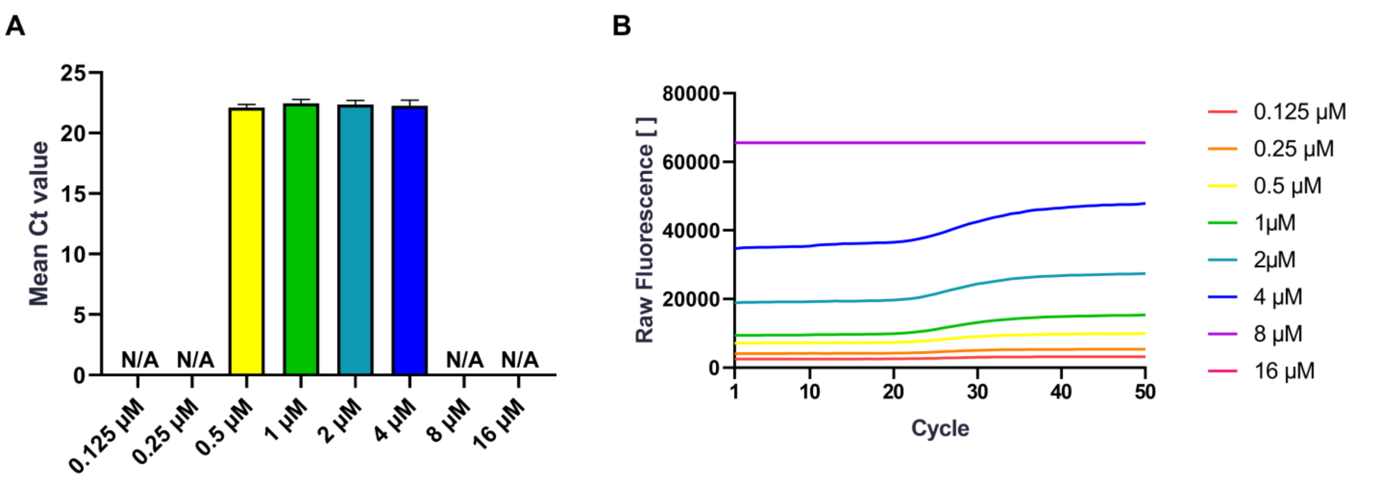

Figure S10. Optimization of the probe-concentration for the detection of VZV using concentrations from 0.125 µM to 16 µM. The concentration for the forward and reverse primers was 1.8 µM for every dilution. (A) Plot of the mean Ct values of duplicates as a function of probe concentration; The error bars correspond to the standard deviation of the duplicates. (B) Plot of the fluorescence curves as a function of the PCR cycle. N/A: No amplification.

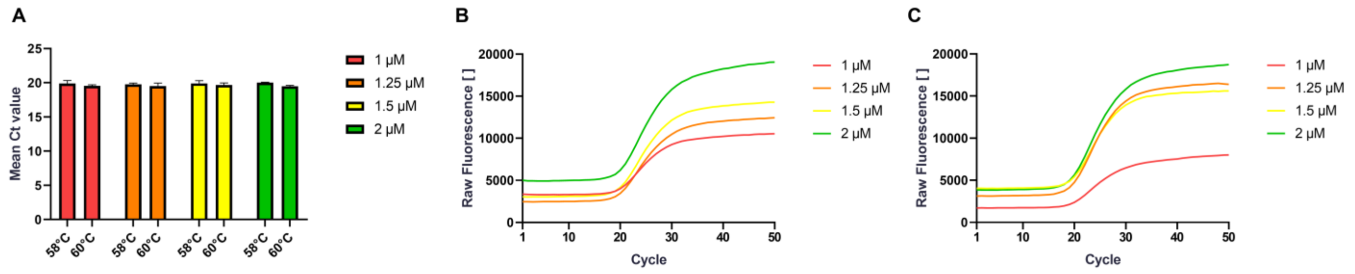

Figure S11. Further optimization of the probe concentration for the detection of VZV for concentrations from 1 µM to 2 µM at two annealing temperatures 58°C and 60°C. (A) Comparison of the mean Ct values for the different probe concentrations at the two annealing temperatures. The error bars correspond to the standard deviation of duplicates. (B) Plot of the fluorescence curves as a function of the PCR cycle for 58°C. (C) Representation of the fluorescence curves as a function of the PCR cycle for 60°C.

Tabelle S1: List of all sequences that were used to generate the consensus sequences for HSV-1, HSV-2, CMV and VZV.

Accession-Nr.	Virus
FV537007.1	HSV-1
FV537008.1	HSV-1
FV537009.1	HSV-1
HM585496.2	HSV-1
HM585497.2	HSV-1
HM585498.2	HSV-1
HM585499.2	HSV-1
HM585500.2	HSV-1
HM585501.2	HSV-1

HM585502.2	HSV-1
HM585503.2	HSV-1
HM585504.2	HSV-1
HM585505.2	HSV-1
HM585506.2	HSV-1
HM585507.2	HSV-1
HM585508.2	HSV-1
HM585509.2	HSV-1
HM585511.2	HSV-1
HM585512.2	HSV-1
HM585513.2	HSV-1
HM585514.2	HSV-1
HM585515.2	HSV-1
JN555585.1	HSV-1
KJ847330.1	HSV-1
KR011285.1	HSV-1
KT780616.1	HSV-1
KX265036.1	HSV-1
KX265043.1	HSV-1
KX265045.1	HSV-1
KX424525.1	HSV-1
LT594105.1	HSV-1
LT594106.1	HSV-1
LT594107.1	HSV-1
LT594108.1	HSV-1
LT594109.1	HSV-1
LT594110.1	HSV-1
LT594111.1	HSV-1
LT594112.1	HSV-1
LT594192.1	HSV-1
LT594457.1	HSV-1
MF156584.1	HSV-1
MG646679.1	HSV-1
MG999842.1	HSV-1
MG999844.1	HSV-1
MG999856.1	HSV-1
MG999860.1	HSV-1
MG999864.1	HSV-1
MG999870.1	HSV-1
MH102298.1	HSV-1
MH160361.1	HSV-1
MH160365.1	HSV-1
MH160367.1	HSV-1

MH160375.1	HSV-1
MH813981.1	HSV-1
MH813989.1	HSV-1
MH813994.1	HSV-1
MH925774.1	HSV-1
MH999840.1	HSV-1
MH999844.1	HSV-1
MN136523.1	HSV-1
MN159376.1	HSV-1
MN159377.1	HSV-1
MN159378.1	HSV-1
MN159380.1	HSV-1
MN401201.1	HSV-1
MT876428.1	HSV-1
NC_001806.2	HSV-1
OL638991.1	HSV-1
ON007144.1	HSV-1
ON007145.1	HSV-1
ON007156.1	HSV-1
ON152715.1	HSV-1
ON513441.1	HSV-1
ON960058.1	HSV-1
ON960059.1	HSV-1
OQ102003.1	HSV-1
OQ658624.1	HSV-1
OQ724957.1	HSV-1
OR771688.1	HSV-1
OR833070.1	HSV-1
LT576866.1	HSV-1
LT576867.1	HSV-1
LT576868.1	HSV-1
LT576869.1	HSV-1
LT576870.1	HSV-1
LT576871.1	HSV-1
LT608134.1	HSV-1
AB618031.1	HSV-1
MN136524.1	HSV-1
JN561323.2	HSV-2
NC_001798.2	HSV-2
KY922722.1	HSV-2
OM370995.1	HSV-2
MF510345.1	HSV-2
MH790610.1	HSV-2

MH790658.1	HSV-2
KY922720.1	HSV-2
MH790576.1	HSV-2
KY922721.1	HSV-2
MF510348.1	HSV-2
MH790582.1	HSV-2
KY922726.1	HSV-2
JX112656.1	HSV-2
FV537011.1	HSV-2
MF510268.1	HSV-2
MF510322.1	HSV-2
MH790666.1	HSV-2
MH790588.1	HSV-2
MH790625.1	HSV-2
MF510325.1	HSV-2
MF510358.1	HSV-2
MH790605.1	HSV-2
MF510277.1	HSV-2
MF510310.1	HSV-2
MF510370.1	HSV-2
KX574904.2	HSV-2
MF510294.1	HSV-2
MF510364.1	HSV-2
MF510344.1	HSV-2
MH790561.1	HSV-2
MH790575.1	HSV-2
MH790631.1	HSV-2
MH790662.1	HSV-2
KX574869.2	HSV-2
MF510327.1	HSV-2
MF564035.1	HSV-2
KP334095.1	HSV-2
KU310668.1	HSV-2
MF510328.1	HSV-2
MH790569.1	HSV-2
MF510285.1	HSV-2
MN187895.1	HSV-2
MH790609.1	HSV-2
MH790667.1	HSV-2
MH790596.1	HSV-2
MH790645.1	HSV-2
MH790597.1	HSV-2
MH790578.1	HSV-2

MH790579.1	HSV-2
MH790628.1	HSV-2
MH790551.1	HSV-2
MH790624.1	HSV-2
KX574873.2	HSV-2
KX574867.2	HSV-2
KX574876.2	HSV-2
MF510301.1	HSV-2
KX574866.2	HSV-2
MF621255.1	HSV-2
KX574878.2	HSV-2
KX574875.2	HSV-2
KX574865.2	HSV-2
KX574877.2	HSV-2
KX574872.2	HSV-2
KX574874.2	HSV-2
KX574860.2	HSV-2
KX574861.2	HSV-2
MH790583.1	HSV-2
MH899846.1	HSV-2
MF510281.1	HSV-2
KX574863.2	HSV-2
KX574871.2	HSV-2
MF621256.1	HSV-2
MF621258.1	HSV-2
KX574893.2	HSV-2
KX574894.2	HSV-2
KR135305.1	HSV-2
MF510366.1	HSV-2
KR135317.1	HSV-2
KR135316.1	HSV-2
KX574906.2	HSV-2
KR135319.1	HSV-2
KR135307.1	HSV-2
KR135318.1	HSV-2
KR135302.1	HSV-2
KR135315.1	HSV-2
KR135301.1	HSV-2
KR135300.1	HSV-2
KR135299.1	HSV-2
KR135303.1	HSV-2
KR135304.1	HSV-2
KX574899.2	HSV-2

MF510324.1	HSV-2
KR135306.1	HSV-2
KX574862.2	HSV-2
KX574864.2	HSV-2
KX574883.2	HSV-2
KX574905.2	HSV-2
KR135320.1	HSV-2
KF781518.1	HSV-2
KX574880.2	HSV-2
KX574879.2	HSV-2
KX574881.2	HSV-2
KX574892.2	HSV-2
KX574902.2	HSV-2
KR135325.1	HSV-2
KR135329.1	HSV-2
KR135323.1	HSV-2
KR135328.1	HSV-2
KR135322.1	HSV-2
KR135324.1	HSV-2
KR135326.1	HSV-2
KR135327.1	HSV-2
NC_006273.2	CMV
MT044477.1	CMV
KT726951.2	CMV
JX512197.1	CMV
MF084223.1	CMV
KT726949.2	CMV
GU179001.1	CMV
OQ466311.1	CMV
MT044481.1	CMV
GQ221975.1	CMV
OQ466312.1	CMV
AY446894.2	CMV
GQ466044.1	CMV
MT044482.1	CMV
KT726950.2	CMV
GU179290.1	CMV
GQ221974.1	CMV
MT044476.1	CMV
KT726947.2	CMV
KJ361971.1	CMV
OU342900.1	CMV
OU342912.1	CMV

KP745691.1	CMV
KP745664.1	CMV
KY490067.1	CMV
KY490068.1	CMV
KY490070.1	CMV
KP745643.1	CMV
KP745642.1	CMV
KP745718.1	CMV
OU342913.1	CMV
KY490069.1	CMV
KY490066.1	CMV
OU342915.1	CMV
KP745685.1	CMV
KY490065.1	CMV
OU342914.1	CMV
KY490063.1	CMV
KY490064.1	CMV
OU342911.1	CMV
OU342918.1	CMV
OU342917.1	CMV
OU342916.1	CMV
OV100760.1	CMV
KJ361964.1	CMV
KJ361967.1	CMV
KJ361966.1	CMV
MW197154.1	CMV
KJ361970.1	CMV
KJ361960.1	CMV
KJ361961.1	CMV
MT070141.1	CMV
MT070142.1	CMV
KJ361959.1	CMV
KJ872542.1	CMV
OV100763.1	CMV
KJ872540.1	CMV
GU179288.1	CMV
OV100762.1	CMV
MT070140.1	CMV
KJ872541.1	CMV
KY490061.1	CMV
MW197156.1	CMV
KP745680.1	CMV

KP745696.1	CMV
KP745727.1	CMV
KP745724.1	CMV
KP745669.1	CMV
KP745652.1	CMV
KP745649.1	CMV
KP745703.1	CMV
KP745676.1	CMV
KP745699.1	CMV
KP745700.1	CMV
KP745648.1	CMV
KP745723.1	CMV
KP745673.1	CMV
KC519321.1	CMV
KP745689.1	CMV
KC519323.1	CMV
KP745722.1	CMV
KP745634.1	CMV
KP745701.1	CMV
KX544840.1	CMV
GQ121041.1	CMV
KX544837.1	CMV
BK000394.5	CMV
KX544831.1	CMV
KF493877.1	CMV
FJ527563.1	CMV
KX101022.1	CMV
KX101023.1	CMV
FJ616285.1	CMV
KX544834.1	CMV
KX544835.1	CMV
KX544833.1	CMV
KX544832.1	CMV
KF021605.1	CMV
MF783091.1	CMV
KX544841.1	CMV
KY002201.1	CMV
MN274568.2	CMV
KX101024.1	CMV
MW980585.1	CMV
KU550089.1	CMV
KU550088.1	CMV
OK000912.1	CMV

OK000911.1	CMV
KU550090.1	CMV
KT959235.1	CMV
KU550087.1	CMV
KJ361951.1	CMV
MT649468.1	CMV
KJ361956.1	CMV
KY490086.1	CMV
JX512202.1	CMV
JX512203.1	CMV
JX512206.1	CMV
KY490073.1	CMV
MT044479.1	CMV
KJ361952.1	CMV
JX512200.1	CMV
JX512205.1	CMV
KY490082.1	CMV
KJ361957.1	CMV
MT649470.1	CMV
KJ361948.1	CMV
KY490087.1	CMV
KJ361949.1	CMV
KY123649.1	CMV
MW528459.1	CMV
KT634296.1	CMV
MT044480.1	CMV
MT044485.1	CMV
KP973639.1	CMV
KU221094.1	CMV
KP973642.1	CMV
KU221090.1	CMV
KP973626.1	CMV
KM192302.1	CMV
AY315197.2	CMV
MN920393.1	CMV
KU221092.1	CMV
KU221091.1	CMV
KP973640.1	CMV
KP973632.1	CMV
KP973637.1	CMV
KP973636.1	CMV
KU221098.1	CMV
KM192298.1	CMV

KP973634.1	CMV
KU221095.1	CMV
KM192299.1	CMV
KU221093.1	CMV
MK290743.1	CMV
MK290742.1	CMV
MK290744.1	CMV
MK422176.1	CMV
MF084224.1	CMV
KR534199.1	CMV
KR534198.1	CMV
KR534210.1	CMV
KR534200.1	CMV
KR534197.1	CMV
KR534202.1	CMV
KR534206.1	CMV
KR534208.1	CMV
KR534203.1	CMV
KR534209.1	CMV
KR534204.1	CMV
KR534212.1	CMV
KR534196.1	CMV
KR534211.1	CMV
KR534205.1	CMV
KR534213.1	CMV
KR534201.1	CMV
KR534207.1	CMV
KT726945.2	CMV
KJ426589.1	CMV
HQ380895.1	CMV
X04370.1	VZV
HW534067.1	VZV
DQ479956.1	VZV
DQ008355.1	VZV
KF558373.1	VZV
FV537017.1	VZV
X04370.1	VZV
DQ479955.1	VZV
DQ479953.1	VZV
DQ479962.1	VZV
NC_001348.1	VZV
KF811485.1	VZV
DQ479959.1	VZV

AY548171.1	VZV
DQ479961.1	VZV
DQ479958.1	VZV
DQ479957.1	VZV
AB097932.1	VZV
DQ008354.1	VZV
FV537016.1	VZV
DQ479954.1	VZV
MH499469.1	VZV
OQ835722.1	VZV
MH499466.1	VZV
MH499467.1	VZV
MH499468.1	VZV
OQ835719.1	VZV
OQ835716.1	VZV
OQ723679.1	VZV
OQ916049.1	VZV
OQ916050.1	VZV
OQ709130.1	VZV
OQ835720.1	VZV
OQ709131.1	VZV
OQ835718.1	VZV
OQ723678.1	VZV
OQ718929.1	VZV
OQ427944.1	VZV
OQ709132.1	VZV
OQ835721.1	VZV
OQ454913.1	VZV
ON023029.1	VZV
OL311042.1	VZV
MK531557.1	VZV
KC847290.1	VZV
MF898328.1	VZV
MW316406.1	VZV
MW545807.1	VZV
MT370830.1	VZV
KU926313.1	VZV
MT370828.1	VZV
MF004348.1	VZV
MW545806.1	VZV
KU926319.1	VZV
KU926315.1	VZV
KU926312.1	VZV

MT370827.1	VZV
KU926311.1	VZV
MT370829.1	VZV
KJ808816.1	VZV
KU926318.1	VZV
MT370825.1	VZV
MW545808.1	VZV
MT370826.1	VZV
KJ767491.1	VZV
KU926320.1	VZV
KU926321.1	VZV
MH709327.1	VZV
MH709330.1	VZV
MH709338.1	VZV
MH709350.1	VZV
MH709320.1	VZV
MH709348.1	VZV
MH709310.1	VZV
MH709357.1	VZV
MH709341.1	VZV
MH709344.1	VZV
MH709374.1	VZV
MH709343.1	VZV
MH709313.1	VZV
MH709377.1	VZV
KF558390.1	VZV
MH709372.1	VZV
DQ674250.1	VZV
MH709364.1	VZV
KF558377.1	VZV
MH709340.1	VZV
KP771909.1	VZV
MG764310.1	VZV
KP771908.1	VZV
KF853226.1	VZV
MG764308.1	VZV
KP771906.1	VZV
KP771889.1	VZV
KP771912.1	VZV
KF558385.1	VZV
KP771910.1	VZV
KF853229.1	VZV
KP771905.1	VZV

KX262865.1	VZV
KF558386.1	VZV
KP771890.1	VZV
KF853231.1	VZV
KF853228.1	VZV
MG764312.1	VZV
KF558371.1	VZV
KP771911.1	VZV
KP771896.1	VZV
KP771902.1	VZV
KP771895.1	VZV
KP771891.1	VZV
KP771903.1	VZV
KP771898.1	VZV
KP771894.1	VZV
KP771899.1	VZV
KP771901.1	VZV
KP771897.1	VZV
KP771900.1	VZV
KP771892.1	VZV
KP771893.1	VZV
KP771914.1	VZV
JN704705.1	VZV
JN704693.1	VZV
JN704708.1	VZV
KP771915.1	VZV
JN704706.1	VZV
JN704694.1	VZV
JN704702.1	VZV
JN704696.1	VZV
JN704690.1	VZV
JN704692.1	VZV
JN704691.1	VZV
AJ871403.1	VZV
JN704695.1	VZV
JN704699.1	VZV
JN704710.1	VZV
JN704707.1	VZV
JN704697.1	VZV
JN704703.1	VZV
JN704701.1	VZV
JN704709.1	VZV
KP771918.1	VZV

KP771922.1	VZV
KP771919.1	VZV
KP771921.1	VZV
KP771923.1	VZV
KP771924.1	VZV
KM355712.1	VZV
KM355707.1	VZV
KM355697.1	VZV
KM355696.1	VZV
KM355716.1	VZV
KM355709.1	VZV
KM355715.1	VZV
KM355701.1	VZV
KM355708.1	VZV
KM355714.1	VZV
KM355700.1	VZV
KM355702.1	VZV
KM355717.1	VZV
KM355703.1	VZV
KM355713.1	VZV
KM355710.1	VZV
KM355718.1	VZV
KM355699.1	VZV
KM355704.1	VZV
KM355706.1	VZV
KC112914.1	VZV
EU154348.1	VZV
DQ452050.1	VZV
